# Prevalence of acute kidney injury and its characteristics among neonates with suspected sepsis in a tertiary hospital in Kenya

**DOI:** 10.4314/ahs.v23i1.75

**Published:** 2023-03

**Authors:** Catherine Munyendo, Bashir Admani, Patrick Mburugu, Justus Simba, Bernadine Lusweti, Naomi Gachara, Ahmed Laving

**Affiliations:** 1 University of Nairobi, Department of Paediatrics and Child Health; 2 Jomo Kenyatta University of Agriculture and Technology, Department of Child Health and Paediatrics; 3 Kenyatta National Hospital, Paediatrics Department; 4 Thika Level Five Hospital, Paediatrics Department

**Keywords:** Acute Kidney Injury, Neonates, Neonatal Sepsis

## Abstract

**Introduction:**

Unique aspects of neonatal renal physiology enhance the occurrence of Acute Kidney Injury (AKI) as a complication of neonatal sepsis. The study sought to determine prevalence of AKI and its characteristics in neonates with suspected sepsis.

**Methods:**

A cross-sectional study was conducted at Kenyatta National Hospital among neonates aged 0-28 days. AKI was defined as serum creatinine of more than 100µmmol/l.

**Results:**

Among 332 neonates included 120 had AKI giving a prevalence of 36.1% (95% CI 31 to 41.6). Based on RIFLE criteria the commonest AKI presentation was Failure 72 (62.6%, 95% CI 53.6 to 71.6), followed by Injury 26 (22.6%, 95% CI 14.8 to 30.4) and then Risk 17 (14.8%, 95% CI 8.2 to 21.3). AKI was more common in neonates with suspected late onset sepsis (p=0.004). Maternal fever in the preceding week to delivery and presence of either puerperal sepsis or post-partum hemorrhage were significantly associated with severe AKI (p=0.004 and p=0.038).

**Conclusion:**

Prevalence of AKI was high; those with suspected late onset sepsis were more likely to develop AKI compared to early onset sepsis. Presence of maternal fever preceding delivery and presence of either puerperal sepsis or postpartum hemorrhage were associated with severe forms of AKI.

## Introduction

Acute Kidney Injury (AKI) is defined as a sudden decline in renal function with inability to excrete nitrogenous wastes. Sepsis is a leading cause of neonatal mortality accounting for 400,000 deaths globally per annum 1. The Kenya Demographic Health Survey 2014 reported a neonatal mortality rate of 22 deaths per 1000 live births in Kenya 2. Findings from a study conducted at Kenyatta National Hospital revealed neonatal sepsis as a leading cause of death followed by prematurity and perinatal asphyxia that are often complicated by sepsis 3.

Unique aspect of neonatal renal physiology predisposes newborns to develop AKI rapidly. Nephrogenesis begins in the 5th week of gestation and is completed at about 34-35 weeks and therefore preterm newborns and those with IUGR have increased susceptibility AKI. The Renin Angiotensin System (RAS) is critical in the normal renal development and renal blood flow in that it vasoconstricts both the afferent and efferent arterioles while the prostaglandins mediate vasodilatation. Dysregulation counter regulatory hormones often occurs in critically ill neonates leading to a rapid decline in glomerular function. This can be worsened by medications such as NSAIDS and nephrotoxic antibiotics like the aminoglycosides frequently used to manage sepsis 4.

Upon the cessation of the fetal circulation, renal blood flow increases progressively from 6% of the total blood volume at birth to 20% by the 6th week of life. During this transitional period, any insult that causes hypoperfusion predisposes to rapid development of AKI. The GFR (Glomerular Filtration Rate) at birth is about 10 to 20ml/min/1.73m^2^ steadily rises to the adult GFR by the 2nd year of life. Any conditions resulting in renal ischemia further lowers the GFR in this critical neonatal period. Tubular function is also immature with decreased concentrating ability and thus poor handling of water and electrolytes 5.

Inflammatory responses that occur during sepsis induce adaptive changes to the tubular epithelial cells to minimize energy demands and enhance survival resulting in reduced kidney function 6.

Overall sepsis is characterized by a cascade of adaptive and maladaptive cellular mechanisms which potentiate each other to give rise to AKI. Sepsis causes significant alteration in microvascular blood flow in the entire body, including the kidneys. The hallmark of microvascular dysfunction is increase in heterogeneity in distribution of blood flowing in the capillaries, resulting in hypoperfusion and hypoxia that worsens damage caused by inflammation^7^. There is evidence that AKI does occur early in the presence of sepsis. Evaluating its prevalence at the time when a clinical diagnosis of suspected sepsis is being made, aids in making appropriate interventions in the management of these neonates.

## Methodology

### Study Design

A hospital based cross sectional study was conducted to determine the prevalence of acute kidney injury and its characteristics among neonates aged 0-28 days admitted with suspected neonatal sepsis at Kenyatta National Hospital.

### Study procedures

Between August and December 2016 newborns admitted with suspected neonatal sepsis were identified from the paediatric wards. In this study suspected neonatal sepsis was considered when at least two of the general symptoms of fever or hypothermia, lethargy, poor feeding and one or more of the systemic symptoms: Respiratory symptoms include tachypnea, grunting and intercostal retractions. Cardiovascular symptoms consist of reduced capillary refill, hypotension, cyanosis, tachycardia or bradycardia. Central nervous system symptoms consist of irritability, bulging fontanelle and altered muscle tone. Suspected sepsis was utilized due to challenges in laboratory diagnosis in our set up.

Demographic data was collected using a standardized questionnaire. Consecutive sampling technique was used. Neonates with suspected sepsis aged 0-28 days of life and parent/guardian consented to participate in the study were included. Those with confirmed congenital renal abnormalities, those with AKI post-surgery and those admitted before day 3 of life were excluded. Creatinine levels for newborns with suspected neonatal sepsis were assessed after day 3 of life. AKI was defined as serum creatinine level above 100µmmols. The neonate's urine output was then monitored for 24 hours from the time of diagnosis of AKI by either urine collection bags or a urinary catheter. The neonatal RIFLE criteria were then used to classify the neonates' severity of AKI.

### IRB Approval

The study was approved by Kenyatta National Hospital/University of Nairobi Ethical Review Committee (ERC) and permission for data collection was sought from Kenyatta National Hospital.

### Data Management and Statistical Analysis

Data were collected and entered into a password-protected MS-Access database. A preliminary data analysis was done to identify extreme values and inconsistencies. Data entry was done and exported to SPSS version 21.0 for analysis.

Continuous values were summarized as means, medians, standard deviation and interquartile ranges. Discrete values were summarized using frequencies and percentages. Categorical variables were compared using chi-square test. A P value <0.05 was considered significant. The associations between social demographics characteristics, risk factors and presence of AKI were measured by Odds Ratios (OR).

## Results

### Characteristics of the neonates with AKI

A total of 332 newborns with suspected sepsis were included. There were 68 (56.7%) males, giving a male-to-female ratio of approximately 4:3. All the neonates were delivered at term (mean gestational age 39.1 weeks, SD ± 1.6), with 111 (92.5%) having a normal birth weight. The mean birth weight was 3216 grams (SD ± 513). Approximately one-quarter (24.9%) of the neonates had early onset neonatal sepsis. The mean age of the neonates with suspected sepsis who were diagnosed with AKI was 11.2 days (SD ± 5.6). (This is shown in table 1)

**Table 1:** Characteristics of neonates with AKI

Characteristic	Frequency n = 120	Percent %	Mean
Male	68	56.7%	
Mean age in days ± SD			11.2(± 5.6)
Early neonatal period(>7days)	29	24.6%	
Late neonatal period (>7days)	89	75.4%	
Mean birth weight ± SD			3216(±513)
Low birth weight	9	7.5%	
Normal birth weight	111	92.5%	
Mean gestation in weeks ± SD			39.1(± 1.6)
Term births	120	100%	

### Prevalence of AKI in suspected neonatal sepsis

Out of the 332 neonates admitted a total of 120 had AKI giving a prevalence of 36.1% (95% CI 31 to 41.6). The most common AKI presentation was Failure 72 (62.6%; 95% CI 53.6 to 71.6), followed by Injury 26 (22.6%; 95% CI 14.8 to 30.4), 17 (14.8%; 95% CI 8.2 to 21.3) children were classified as Risk.

### Renal functions of neonates with AKI

The 24-hourly urinary output ranged from 3mls to 286mls with a mean output of 123.2 ± 67.4. The mean output in milliliters per kilogram body weight was 1.8 ± 1.1. Serum creatinine levels ranged from 118 to 1027 µmmol/ l with a mean serum creatinine level of 393.3 ±200.5.

### Neonatal characteristics and AKI severity

There was a significant association between AKI and age of neonate (ANOVA p = 0.04). Neonates classified as having AKI risk were on average aged 8.3 days compared to those with injury (mean = 12.8 days ± 7.1) and those in failure (mean = 11.2 days ± 5.2). (This is shown in table 2).

**Table 2:** Association between AKI severity and neonatal characteristics

	Risk	Injury	Failure	
Characteristics	Mean ± SD	Mean ± SD	Mean ± SD	P
Mean age in days	8.3 ± 4.6	12.8 ± 7.1	11.2 ± 5.2	0.04
Mean gestation (weeks)	39.1 ± 1.2	39.2 ± 1.5	39 ± 1.7	0.8
Mean birth weight (grams)	3229 ± 522	3263 ± 512	3181 ± 512	0.7

The difference in age between neonates at risk and those with injury was statistically significant (Bonferroni P = 0.035) while that between injury and failure or risk and failure was not significant. Mean gestational age (p = 0.823) and birth weight (p = 0.767) were not significantly associated with AKI.

### Maternal characteristics

The modal age of mothers was 20-29 years, 76 (60.8%), with a mean age of 26 years (± 5.9). Most of the mothers 68 (56.6%) were primigravid, and reported that they were married,85 (70.8%). Most of the neonates we saw were referrals having been delivered at other facilities, 82 (72.8%). (This is summarized in Table 3)

**Table 3:** Summary of maternal characteristics

Characteristics	Frequency n=120	Percent %
**Maternal Age**		
< 20 years	12	10%
21–29 years	73	60.8%
>30 years	25	20.8%
**Occupation**		
Salaried/self employed	53	48%
Unemployed/casual	62	57.1%
**Marital Status**		
Married	85	70.8%
Single	35	29.1%
**Level of Education**		
Primary education	28	23.3%
Secondary education	58	48.3%
Tertiary education	29	26%
**Place of Delivery**		
KNH delivery	27	22.5%
Delivered in other facility	82	72.8%
**Mode of Delivery**		
Vaginal delivery	75	62.5%
Caesarean section delivery	32	28.5%
**Primiparity**	68	56.6%
**Maternal fever**	100	89.8%
**Meconium-stained liquor**	71	63.8
**Post-partum hemorrhage**	5	4.1%
**Postpartum sepsis**	5	4.1%
**Duration of stay < 48hrs**	68	60.8%
**History of UTI**	1	0.83%

### Association between maternal characteristics and AKI severity

There was no significant association between maternal age, primiparity, level of education, place of delivery and AKI severity p>0.05. The presence of maternal fever in the week preceding delivery was associated with AKI severity in the neonates p = 0.04. Similarly, the presence of a post-partum complications sepsis and hemorrhage was significantly associated with AKI severity p = 0.03. The mothers of the neonates with AKI had a short duration of hospital stay post-partum of less than < 48 hours – 68 (60.8%). (This is shown in table 4)

**Table 4:** Association between maternal characteristics and AKI severity

Characteristics	Risk n = 17 (%)	Injury n = 26 (%)	Failure n = 72 (%)	P
**Maternal Age**				
20 years	2 (11.7)	2 (7.6)	8 (61.5)	0.5
20–29 years	10 (58.8)	15 (57.6)	48 (63.2)	
30 years +	5 (29.4)	9 (52.9)	12 (44.4)	
**Parity**				
Primiparity	13 (76.7)	11 (42.3)	44 (61.1)	0.07
Multi parity	4 (23.5)	15 (57.6)	28 (38.8)	
**Marital Status**				
Single	5 (29.4)	4 (15.3)	21 (29.1)	0.6
Married	12 (70.5)	22 (84.6)	51 (70.8)	
**Occupation**				
Salaried/informal employment	9 (52.9)	14 (53.8)	30 (41.6)	0.6
Casual worker/unemployed	8 (47)	12 (46.1)	42 (58.3)	
**Level of Education**				
Primary	5 (29.4)	9 (34.6)	14 (19.4)	0.4
Secondary	9 (52.9)	9 (34.6)	40 (55.5)	
Tertiary	3 (17.6)	8 (30.7)	18 (25)	
**Place of Delivery**				
KNH	6 (35.2)	7 (26.9)	14 (19.4)	0.3
Other facility	10 (58.8)	19 (73)	57 (79.1)	
**Mode of Delivery**				
Vaginal	11 (64.7)	16 (61.5)	50 (69.4)	0.68
CS	3 (17.6)	8 (30.7)	21 (29.1)	
**Maternal Fever**	16 (94.1)	23 (88.4))	61 (84.7)	0.04
**Liquor**				
Clear liquor	5 (29.4)	10 (38.4)	29 (40.2)	0.7
Meconium-stained liquor	12 (70.5)	16 (61.5)	43 (59.7)	
**Complications**				
Postpartum hemorrhage	1 (0.05)	2 (0.07)	2 (0.02)	0.03
Postpartum sepsis	0 (0.0)	0 (0.0)	5 (100.0)	
**Duration of stay**				
Less than 24hrs	4 (18.2)	6 (27.3)	11 (50.0)	0.3
24–48hrs	6 (11.8)	9 (17.6)	32 (62.7)	
>72hrs	6 (15.4)	9 (23.1)	24 (61.5)	

## Discussion

We found an AKI prevalence of 36.1 % (95% CI 31 to 41.6) in neonates admitted with suspected sepsis. These results compare closely with other studies conducted to establish AKI prevalence in neonatal sepsis. Pradan et al in a study in India on AKI and neonatal sepsis found a 27.5% prevalence of AKI, Vachvanichsanong et al in a study in Thailand found a prevalence of 30.9% while Mathur et al in their study in India found an AKI prevalence of 26% 8–10.

Most of the neonates in our study were in failure. These results are contrary to a study by Mohkan et al in Tehran-Iran where the AKI prevalence in their study was 77% with 43% of the neonates in the risk category, 51% in the injury category and 6% in the failure category 11. The assessment of the renal function of the neonates with suspected sepsis revealed that AKI was predominantly non-oliguric with the mean urine output at 1.8ml per kg/hour. This finding is consistent with other studies on neonatal AKI 9,12.

There were 68(56.7%) males and 52(43.3%) females among the 120 neonates with AKI; this give a male to female ratio of 4:3. Bansal et al in a case control study in India found that the male gender and sepsis were the only variables that had a significant association with AKI. The male gender had an odds ratio of (OR=2.84) (CI=1.12-7.21) and sepsis odds ratio of (OR=14.6) (CI 4.5-46.46)^13^. Youssef et al in a prospective study conducted in neonates admitted in NICU in a children's hospital in Egypt found an AKI prevalence of 10.8%, with a male sex predominance of 1.3:1 13.

Our study found that AKI occurred more in neonates with late onset neonatal sepsis. This is different from a study by Holda et al who in their study in India found that AKI occurred more frequent in neonates who had early onset neonatal sepsis at 54.8% and 45.2% for late onset sepsis contrasting 14. In our study this might be explained by a delay by parents and guardians in seeking healthcare services.

The maternal demographic factors under investigation were found to have no significant association with AKI in the neonates with suspected sepsis. Primiparity and presence of maternal fever within a week of delivery was found to be associated with AKI. This finding is similar to that found in a study by Cataldi et al in Italy on potential risks for acute renal failure in preterm neonates that found peripartum sepsis and exposure to antibiotics in the immediate peripartum period significantly associated with AKI (p=0.004). In the study by this study by Cataldi et al, primiparity was not significantly associated with AKI (p=0.78) 15.

Limitations of this study is the cross-sectional design hence we were not able to conclusively generate correlates on risk factors and determine a true causal association.

## Conclusion

Thirty six percent of neonates admitted with suspected sepsis are likely to develop AKI. The severity of AKI using the RIFLE criteria correlates with the severity of sepsis. Neonates with late onset neonatal sepsis are twice more likely to develop AKI compared to those with early onset neonatal sepsis. Male neonates are two times more likely to develop AKI than their female counterparts.

## Recommendations

Clinicians should screen for AKI in all neonates with suspected sepsis by assessment of serum creatinine levels at the earliest opportunity. This will pave way for timely interventions to preserve kidney function. Further studies on neonatal AKI are required to establish outcomes considering the fact that the majority of the neonates with AKI had the severe form of AKI and to evaluate the quality of care offered to these neonates.
